# Longitudinal volume evolution of large and giant intracranial aneurysms following endovascular flow diversion

**DOI:** 10.1016/j.bas.2026.106172

**Published:** 2026-07-23

**Authors:** Sergio García-García, Andreas Ziebart, Mika Niemelä, Jussi Numminen, Rahul Raj

**Affiliations:** a Department of Neurosurgery, Hospital Universitari Germans Trias i Pujol, Barcelona, Spain; b Department of Neurosurgery, Medical University of Innsbruck, Innsbruck, Austria; c Department of Neurosurgery, Helsinki University Hospital and University of Helsinki, Helsinki, Finland; d Department of Radiology, Helsinki University Hospital and University of Helsinki, Helsinki, Finland

**Keywords:** Intracranial aneurysm, Flow diversion, Radiology, Imaging, Outcome

## Abstract

**Introduction:**

Large and giant intracranial aneurysms (IAs) pose significant therapeutic challenges due to their complex morphology and high rupture risk. Flow diverters (FD) promote intrasaccular thrombosis, facilitating occlusion. However, the temporal volumetric evolution of FD-treated large and giant IAs remains variable and poorly understood. Here, we sought to assess the temporal volumetric evolution of large and giant aneurysms treated with FD.

**Methods:**

Retrospective single-center study (2018–2022) of aneurysms ≥15 mm treated with FD, with late MRI follow-up. Demographic, morphological and radiological variables (total/patent volumes on T2, TOF hyperintensity, FLAIR edema, T1 contrast enhancement) were collected and analyzed together with complications and occlusion status.

**Results:**

Twenty-four patients (median age 64 years, 54% female) were analyzed (saccular 75%, unruptured 96%, anterior circulation 88%). Median follow-up was 41.3 months by MRI and 24.7 months by angiography. Mortality was 17% (4/24), including three fatal post-treatment aneurysm ruptures. After FD treatment, total aneurysm volume did not significantly decrease (−0.86 cm^3^, p = 0.19) but patent aneurysm volume significantly reduced (−1.15 cm^3^, p = 0.001), although the individual volume change was heterogenous. Complete occlusion rate was 79%. Aneurysm occlusion was associated with narrower necks (7 vs. 13 mm, p = 0.023) and smaller initial total aneurysm volumes (2.6 vs. 10.6 cm^3^, p = 0.04).

**Conclusions:**

In large and giant aneurysms treated with FDs, despite high occlusion rates and decreased patent volumes, aneurysm total volume is not similarly reduced. This suggests that the effect of flow diversion is driven mainly by intrasaccular flow cessation and occlusion rather than by measurable volume reduction. Larger studies are needed to confirm our findings.

## Highlights

• Patent aneurysm volume is nearly eliminated after flow diversion.
• Total sac volume shows no significant reduction and may transiently enlarge.
• Smaller initial volume and narrower neck are associated to complete occlusion.
• High angiographic occlusion rates do not ensure sac shrinkage.
• Our findings support individualized follow-up with serial MRI and DSA monitoring.

## 1 Introduction

Large and giant intracranial aneurysms represent a significant challenge in neurosurgical practice due to their size, complex morphology, high risk of rupture and treatment related morbidity (Orz et al., 2000; Suzuki et al., 2020; Tominaga, 2022). Clinically, these aneurysms may rupture, leading to subarachnoid hemorrhage, or remain unruptured causing neurological symptoms due to their mass effect on adjacent brain parenchyma and cranial nerves, as well as ischemic events related to intrasaccular thrombosis and distal embolism (Fried and Yballe, 1972; Choi and David, 2003; Ferns et al., 2010; Brown et al., 2016).

Treatment aims to prevent aneurysmal rupture and alleviate neurological symptoms related to mass effect (Choi and David, 2003; Ferns et al., 2010; Enomoto et al., 2020; Lawton and Spetzler, 1998; Suzuki and Sato, 1980; Wang et al., 2019). Management options include both surgical and endovascular techniques. Microsurgical procedures are technically demanding, often requiring revascularization techniques, are preferred when mass effect symptoms predominate and carry higher risks in large and giant aneurysms (Lawton and Spetzler, 1998; Suzuki and Sato, 1980; Luzzi et al., 2020; Gandhi et al., 2019). Over the past decade, aneurysm treatment has been transformed by the introduction of flow-diverting stents (FD) (Lauzier et al., 2023; Chalouhi et al., 2014; De Vries et al., 2013).

FD effectively reroute hemodynamic flow away from the aneurysm sac, promoting gradual thrombosis within the sac (De Vries et al., 2013; Wakhloo et al., 2015). FDs are naïve to aneurysm's shape or size, which makes them particularly advantageous for aneurysms characterized by wide necks or complex morphologies (Tominaga, 2022; Ferns et al., 2010; Luzzi et al., 2020). The flow diversion process ultimately creates a scaffold for progressive thrombosis, fibrosis, and remodeling, which can lead to notable volume reduction and, in some instances, symptomatic relief (Brown et al., 2016; Szikora et al., 2013; Patel et al., 2015).

However, the post-therapeutic course of large and giant aneurysms remains unknown. The mechanisms underpinning aneurysm involution, and, conversely, those driving potential complications, have not been fully elucidated (Moon et al., 2023). Morphological and volumetric outcomes appear to be influenced by patient characteristics, aneurysm architecture, device specifics, and antithrombotic regimes; yet the interplay between these variables remains poorly defined (Wang et al., 2019; Szikora et al., 2013; Moon et al., 2023; Bender et al., 2020; Kulcsar et al., 2012). In addition, unstable thrombus formation, inflammation, transient aneurysm enlargement, or delayed rupture have all been described as potential adverse outcomes, sometimes resulting in catastrophic consequences (Kulcsar et al., 2011; Frosen et al., 2004; Zhou et al., 2017; Brinjikji et al., 2015). Furthermore, the necessity for dual antiplatelet therapy (DAPT) introduces an additional layer of complexity and clinical risk, particularly in the event of peri-procedural or delayed hemorrhagic complications (Brinjikji et al., 2015).

Flow diversion is an accepted treatment for large and giant intracranial aneurysms that could potentially alleviate compression symptoms through intrasaccular flow disruption and thrombus organization. However, data regarding the volumetric evolution of these lesions remain scarce. The limited understanding of how these mechanisms translate into temporal changes in aneurysm volume underscores the need for further investigation into the pathophysiology and long-term consequences of flow diversion. The present study evaluates longitudinal volumetric changes in large and giant aneurysms (defined as ≥15 mm) treated with flow diversion, and explores their association with morphological, clinical and radiological characteristics.

## 2 Methods

We conducted a single-center, retrospective study including all consecutive patients with large intracranial aneurysms (>15 mm) treated with flow diversion at Helsinki University Hospital. The study was approved by the institutional review board (HUS/313/2022). In accordance with national regulations, informed consent was not required due to the retrospective, observational nature of the research. The study was conducted in compliance with the Strengthening the Reporting of Observational Studies in Epidemiology (STROBE) guidelines (Vandenbroucke et al., 2007).

### 2.1 Study population

We included patients treated with one or more FDs between June 2018 and November 2022 for aneurysms ≥15 mm. We excluded mycotic or blister-type aneurysms. All patients had at least one late follow-up imaging study with both magnetic resonance imaging (MRI) and magnetic resonance angiography (MRA). Aneurysms previously treated by any other method were eligible for inclusion, and no restrictions were applied regarding the manufacturer of the FD device.

Periprocedural antiplatelet management consisted of DAPT with acetylsalicylic acid (ASA) and a P2Y12 inhibitor. During most of the study period, patients received ASA 100 mg/day plus clopidogrel 75 mg/day; during the last years clopidogrel was progressively replaced by prasugrel, at a maintenance dose of 10 mg/day. Before elective procedures, the DAPT was commenced 5-7 days before with platelet function testing (VerifyNow®) at the morning of the intervention. In acute settings, intravenous eptifibatide or abciximab was administered followed by P2Y12 inhibitor and ASA loading and maintenance. After treatment, DAPT is regularly maintained for 6 months.

### 2.2 Variables

We collected demographic variables, cardiovascular risk factors (smoking status and hypertension), symptoms at presentation, morphological aneurysm's features (neck, height, dome and maximum diameter), procedure information (device type, size and number; intraprocedural events or complications) and antiplatelet regime from electronic health care records.

### 2.3 Imaging analysis

We conducted the radiological assessment of follow-up MRIs and MRAs at the following time points: initial (diagnostic); interim follow-up (if available) and final available follow-up (available for all). Two authors conducted the radiological assessments (SGG, AZ). Volumes were measured using the Neuroelements software (Brainlab SE, Munich, Germany). The area of the total aneurysm volume or patent volume were manually drawn on MRI slices with the computer-assisted Smartbrush tool. The resulting 3D reconstruction was used to calculate the volumes in cm^3^. The drawings were manually reviewed and corrected using the automated reconstruction. Additional imaging modalities, such as CT angiography or digital subtraction angiography (DSA), were reviewed when available to complement or reassure volumetric and morphometric analysis. MRI examinations were performed on both 1.5-T and 3.0-T scanners, with slice thickness ranging from 0.7 to 3 mm (TOF 0.7 mm, FLAIR 1 mm, T2 2-3 mm).

We assessed total aneurysm volume in T2; patent aneurysm volume in time-of-flight (TOF) native and gadolinium (Gd) enhanced or T2 if the former was not available (T2 void signal); T1-Gd hyperintensity (intimal enhancement within the flow void); perilesional edema in FLAIR or T2; aneurysm wall gadolinium enhancement.

### 2.4 Outcomes

The primary outcome was reduction in total aneurysm volume on the latest MRI follow-up. Secondary outcomes were patent aneurysm volume on the latest MRI, and aneurysm occlusion assessed by the O'Kelly-Marotta scale (OKM) at the last DSA follow-up (O'Kelly et al., 2010). We also retrospectively assessed the last available functional outcome using the modified Rankin Scale (mRS).

### 2.5 Statistics

Data were collected using Microsoft Excel (Microsoft, Redmond, WA, USA; version 16.16.4) and analyzed in Python (version 3.7) with the Pandas library. Graphical outputs were generated using Matplotlib.

Quantitative variables were summarized as mean ± standard deviations for normally distributed data, or median with interquartile ranges for non-parametric distributed data. Categorical variables were expressed as frequencies and percentages.

Comparisons between groups were performed using Student's t-test or one-way ANOVA for normally distributed variables, and Mann–Whitney *U* test, Wilcoxon signed-rank test (for paired data), or Kruskal–Wallis test for non-normally distributed variables, depending on the number of groups. Associations between categorical variables were analyzed using the Chi-square test or Fisher's exact test, as appropriate.

To evaluate the evolution of aneurysm volume and MRI signal characteristics over time, paired statistical methods were applied. All statistical tests were two-tailed, and results were considered significant at a threshold of *p* < 0.05.

Agreement between the two independent raters was illustrated by Bland–Altman plots for each time point and volumetric parameter. Only complete paired measurements were included in each analysis, with missing observations omitted on a per analysis basis. Mean bias was calculated as the average difference between observers, and the 95% limits of agreement were defined as the mean difference 1.96 standard deviations of the differences.

## 3 Results

A total of 25 patients were treated with FDs between June 2018 and November 2022 with aneurysms ≥15 mm. The follow-up period extended until May 2025. One patient was excluded due to lack of follow-up image. Volume analysis was available for 24 patients in whom aneurysm volume could at least be measured at two time points. Two patients lacked long-term follow-up (>6 months) because they died within the first month after treatment (at 3 days and 2 weeks). The median follow-up from intervention to the interim MRI was 5.5 months (IQR 3.0–17.5), to the latest MRI 41.3 months (IQR 22.2–52.6), and to the latest DSA 24.7 months (IQR 11.7–28.4).

Median age was 64 years and 54% were female patients. One third (33%) of patients had a previous diagnosis of hypertension and the same proportion of patients were active smokers or had a history of smoking (Table 1). Most aneurysms were in the anterior circulation (88%), saccular (75%), unruptured (96%), and had not been treated before (75%). Only one aneurysm was treated acutely after rupture. Half of the aneurysms were incidental findings, while visual symptoms were the most common clinical manifestation (25%). Other presentations included previous subarachnoid hemorrhage, transient leg weakness, hydrocephalus, and hypopituitarism (Supplementary Table 1). Median maximum aneurysm diameter was 19 mm.

**Table 1:** Baseline patient and aneurysm characteristics.

Characteristic	Value (N = 24)
**Patient characteristics**	
Age, years – median (IQR)	64 (56–69)
Female – n (%)	13 (54)
**Hypertension** – **n (%)**	
Yes	8 (33.3)
No	9 (37.5)
Unknown	7 (29.2)
**Smoking – n (%)**	
Yes	8 (33.3)
No	7 (29.2)
Unknown	9 (37.5)
**Symptoms at presentation – n (%)**	
Incidental (asymptomatic)	12 (50.0)
Visual symptoms^a^	6 (25.0)
Unrelated SAH	2 (8.3)
Related SAH	1 (4.2)
Hypopituitarism	1 (4.2)
Hydrocephalus	1 (4.2)
Stroke/TIA	1 (4.2)
Initial mRS^b^ – median (IQR)	1 (0–1)
mRS 0/1/2/5 – n (%)	10 (41.7)/10 (41.7)/3 (12.5)/1 (4.2)
**Aneurysm characteristics**	
**Type – n (%)**	
Saccular	18 (75.0)
Dissecting	4 (16.7)
Fusiform	2 (8.3)
**Location – n (%)**	
ICA, cavernous	11 (45.8)
ICA, communicating	5 (20.8)
ICA, paraophthalmic	3 (12.5)
Basilar	3 (12.5)
ACOM	2 (8.3)
Anterior circulation (total)	21 (87.5)
Previously treated (recurrent) – n (%)	6 (25.0)
Neck, mm – median (IQR)	7.9 (5.9–10.8)
Dome, mm – median (IQR)	15.8 (12.9–21.1)
Height, mm – median (IQR)	17.0 (14.7–21.0)
Maximum diameter, mm – median (IQR)	19.0 (16.0–24.1)

Data are median (interquartile range) or n (%). *Abbreviations:* ACOM, anterior communicating artery; CN, cranial nerve; ICA, internal carotid artery; IQR, interquartile range; mRS, modified Rankin Scale; SAH, subarachnoid hemorrhage.

a Including diplopia (CN3 or CN6 paresis), visual loss, eyelid flickering.

b mRS at the time of the treatment.

Most cases were treated with a single flow diverter (88%), most commonly a Surpass Evolve device (Stryker®; 79%). Antiplatelet therapy comprised aspirin plus clopidogrel in 14 patients (58%), aspirin plus prasugrel in 7 (29%), P2Y12-inhibitor monotherapy in 2 (8%), and intravenous eptifibatide in 1 (4%, acutely ruptured case).

Four patients died during follow-up. In three, the treated aneurysm ruptured after treatment at 3 days, 2 weeks, and 9 months (cases 19, 2, and 22), each causing fatal subarachnoid hemorrhage. The fourth patient died 2.5 years after treatment from respiratory infection and respiratory insufficiency, unrelated to the aneurysm (case 10). One patient suffered an intraprocedural bleeding (case 23) which was controlled without further consequences. An intraprocedural embolus at an A2 branch was suspected in one previously ruptured and coiled aneurysm, but this event was with no clinical worsening (case 14). No other complications associated with the treatment were observed in the rest of the cohort.

Final follow-up functional outcome was good (mRS 0–2) in 20 of 24 patients (83%); the remaining four (17%) died, and among survivors the mRS improved in four, was unchanged in 14, and worsened by one point in two (Table 2).

**Table 2:** Angiographic and clinical outcomes.

Outcome	Value (N = 24)
**Angiographic occlusion (O'Kelly–Marotta grade)**	
At end of procedure — n (B/C/D)	13/9/2
At interim follow-up — n (B/C/D)^a^	1/6/5
At last follow-up — n (B/C/D)^b^	2/1/19
Complete occlusion (grade D) at last follow-up — n (%)	**19 (79.2)**
Time to complete occlusion, months — median (IQR)^c^	7 (6–9)
**Clinical outcome**	
mRS at last follow-up — median (IQR)	0.5 (0–1)
mRS 0/1/2/6 — n (%)	12 (50)/6 (25)/2 (8)/4 (17)
Good outcome (mRS 0–2) — n (%)	20 (83.3)
**mRS change from baseline — n (%)**	
Improved (≥1 point)	4 (16.7)
Unchanged	14 (58.3)
Worsened (≥1 point)	6 (25.0)
**Death** — n (%)	**4 (16.7)**

Data are median (interquartile range) or n (%). Complete occlusion defined as O'Kelly–Marotta grade D. *Abbreviations:* IQR, interquartile range; mRS, modified Rankin Scale.

a Interim angiographic data available for 12 of 24 patients.

b Last-follow-up angiographic data available for 22 of 24 patients; the 2 patients who died within the first 2 months had no follow-up imaging and are counted as not occluded.

c Calculated among the 19 aneurysms achieving complete occlusion; non-occluded aneurysms are excluded.

### 3.1 Aneurysm volume change

**Fig. 1:**
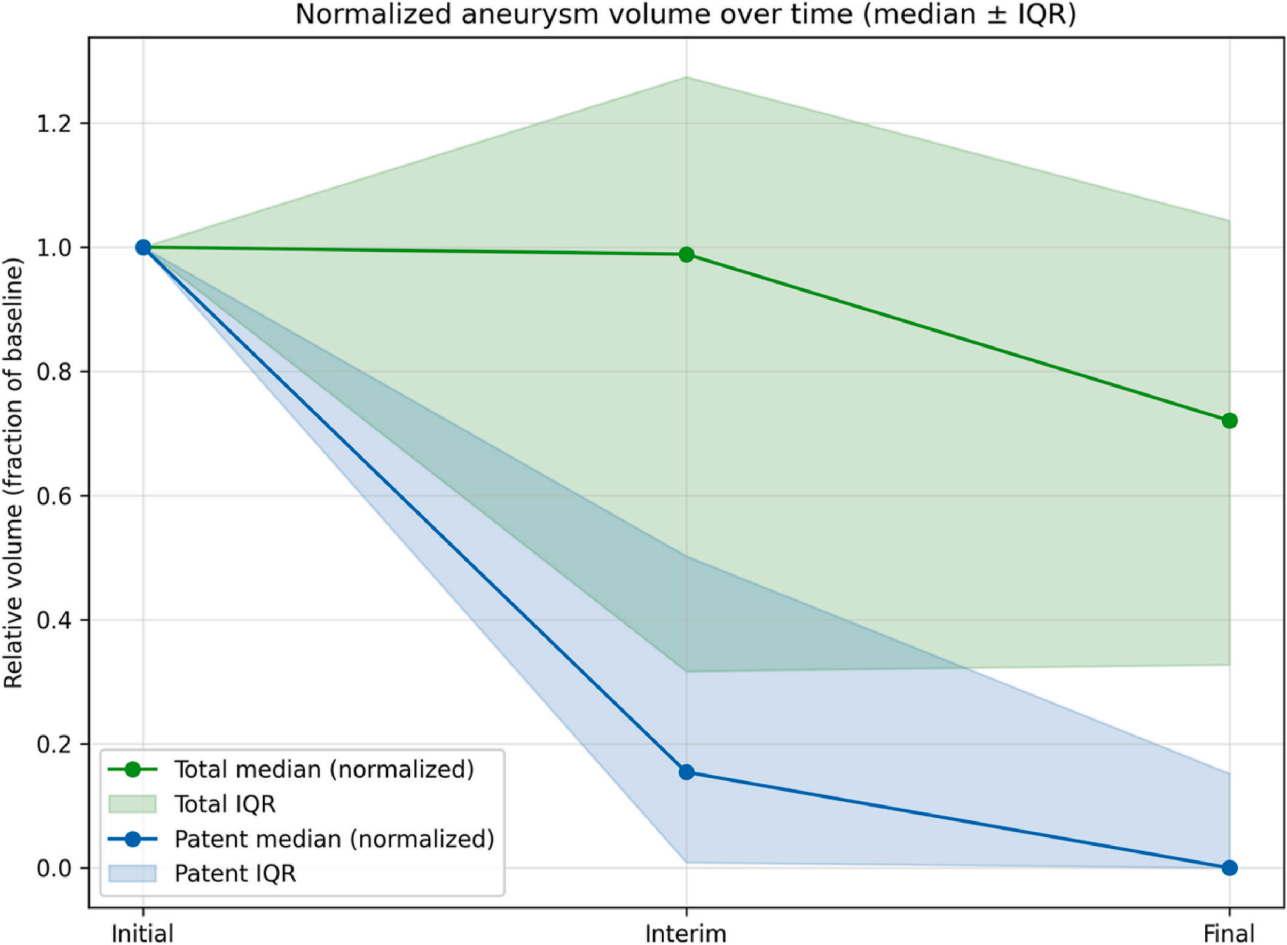
Graphic depicting the volumetric evolution of the analyzed aneurysms: Total (Green) and patent (blue) volumes are represented as a fraction of the baseline.

**Fig. 2:**
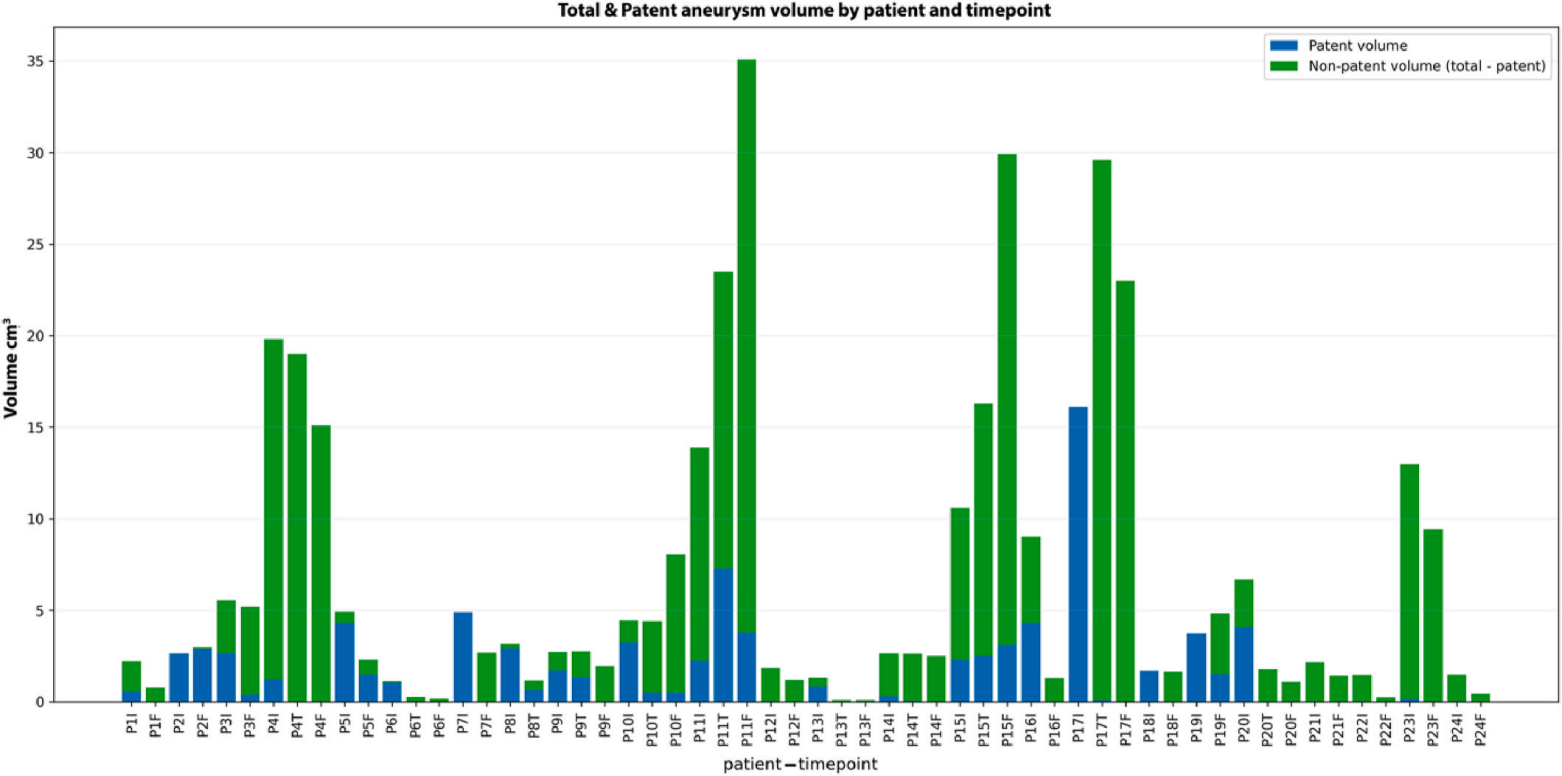
Bar chart of the individual volumetric evolution of every patient at each timepoint. F: Final; I: Initial; P: Patient; T: Interim.

**Fig. 3:**
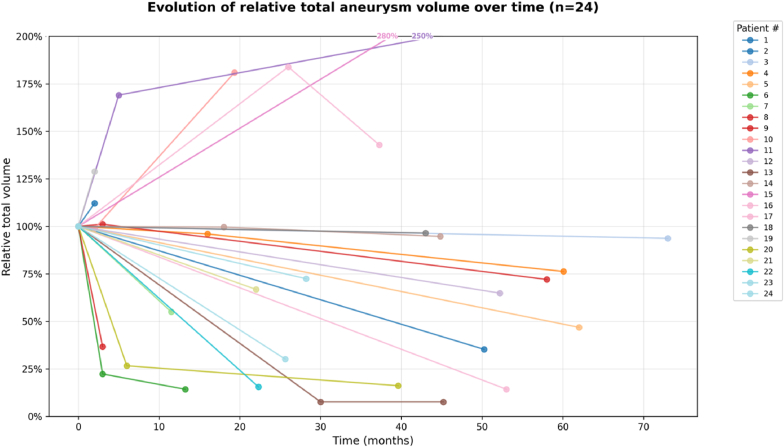
Individual evolution of total volumes over time. Lines ending with a numeric value indicate that the case has been censored at 200% and the real value is represented by the figure.

Total aneurysm volume decreased from baseline to the latest MRI, but the reduction was not statistically significant (median change −0.86 cm^3^, IQR −2.02 to 0.13, p = 0.19), corresponding to a median relative reduction of 28% (IQR −4 to 77%) (Table 3, Fig. 1). Total volume was larger at last follow-up than at baseline in 6 aneurysms (Fig. 2, Fig. 3).

**Table 3:** Aneurysm volumes and follow-up time points.

Volume	Distribution	Value (cm^3^)	Relative value	Median MRI time point
Total initial	Non-normal	3.46 (2.09–7.26)	100%	0 months
Patent initial	Non-normal	1.98 (0.50–3.37)	100%	
Total interim	Non-normal	2.75 (1.47–17.65)	99% (32–127%)	5.5 months (IQR 3.0–17.5)
Patent interim	Non-normal	0.08 (0.00–0.61)	1% (0–21%)	
Total final	Non-normal	1.96 (0.93–6.62)	72% (33–104%)	41.3 months (IQR 22.2–52.6)
Patent final	Non-normal	0.00 (0.00–0.45)	0% (0–15%)	

Data are median (interquartile range). All variables were non-normally distributed. Total volume = thrombus + patent lumen; patent volume = residual contrast-filling lumen. Relative values are expressed as a percentage of the corresponding baseline volume.

**Fig. 4:**
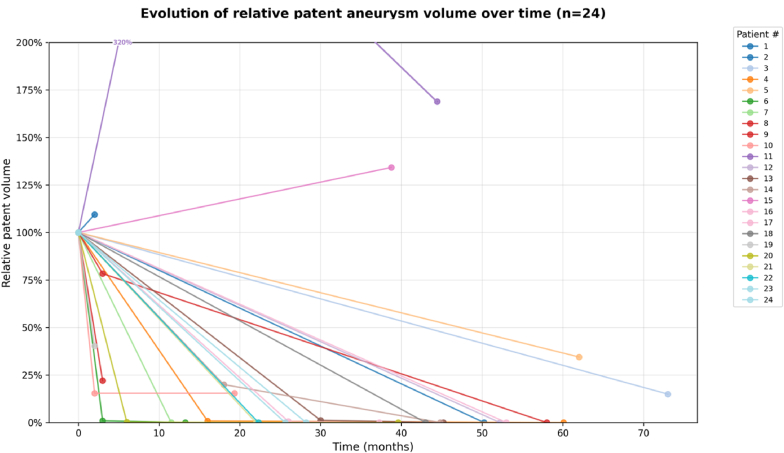
Individual evolution of patent volumes over time. Lines ending with a numeric value indicate that the case has been censored at 200% and the real value is represented by the figure.

Patent aneurysm volume decreased significantly (median change −1.15 cm^3^, IQR −2.50 to −0.01, p < 0.001), reflecting near-complete elimination of the patent lumen (median relative reduction 100%, IQR 85–100%). Patent volume increased only in 3 aneurysms, and final patent volume was at or near zero in most cases (Fig. 4).

Interobserver agreement for patent volume was close at all time points, with small mean differences and narrow limits of agreement (Bland–Altman analysis; **Supplementary Image 1**). A sensitivity analysis excluding the four aneurysms whose patent volume was estimated without TOF imaging did not materially alter the findings: final patent volume remained near zero and the progressive reduction in patent volume was preserved (Supplementary Table 2).

### 3.2 Aneurysm occlusion

**Fig. 5:**
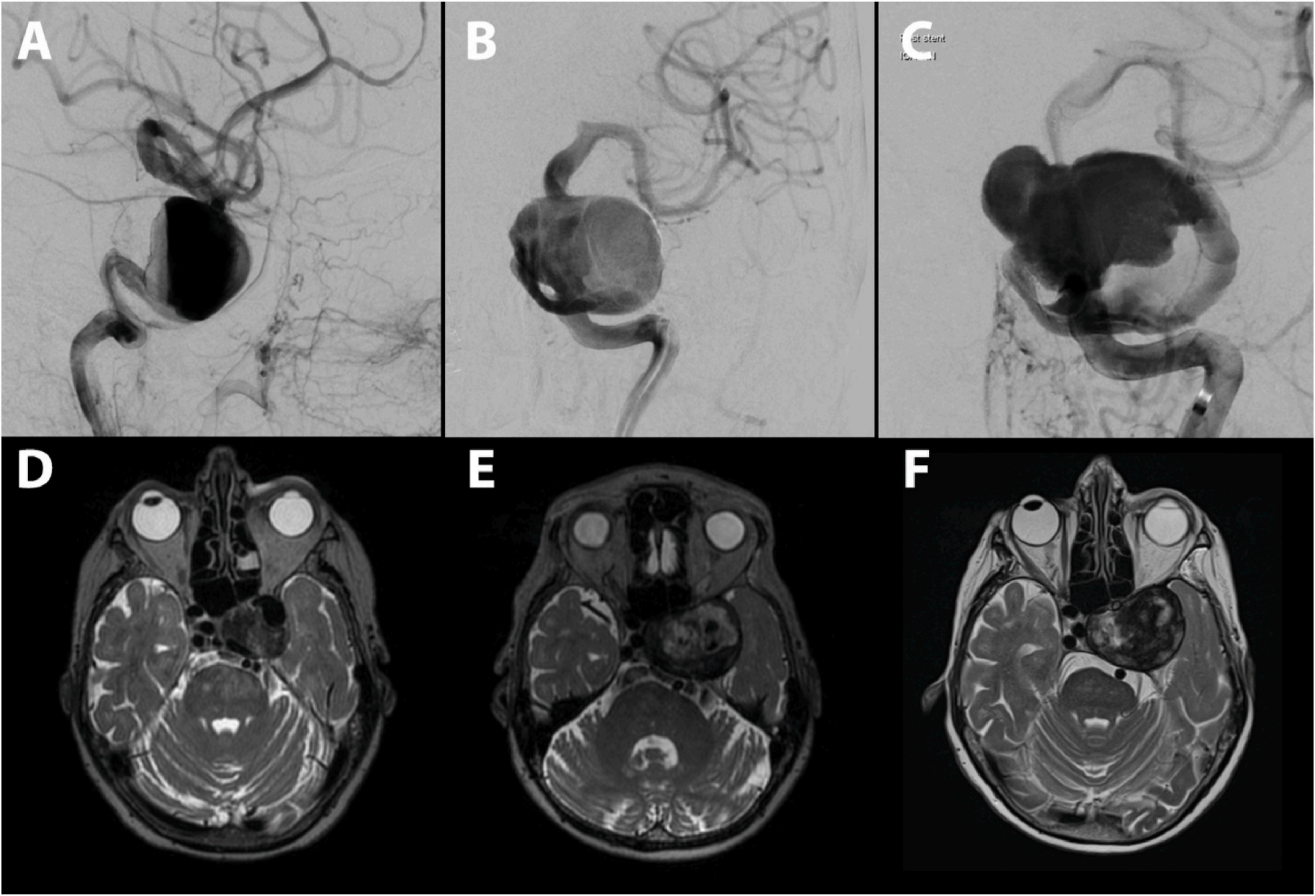
Case 11. Failed occlusion of a giant left Internal Carotid artery (ICA) aneurysm. Lateral (A) and AP (B) diagnostic angiograms of the left ICA. AP angiogram immediately after deploying the flow diverter. Initial (D), Interim (E) and Final MRI- T2 axial views of the patient demonstrating the increasing size of the aneurysm following failed occlusion.

At last follow-up, complete occlusion was achieved in 79% of aneurysms (n = 19/24). Among those that ultimately occluded, 79% (n = 15/19) did so by the first follow-up DSA at 6–9 months (Supplementary Table 3). Occlusion occurred only later, between 24 and 45 months, in 21% (n = 4/19). One aneurysm failed occlusion, a previously coiled recurrent aneurysm (case 3), was retreated with an additional flow diverter in a second procedure but remained unoccluded at last follow-up. A giant ICA aneurysm (case 11; Fig. 5) likewise failed to occlude and progressively enlarged.

Complete occlusion was associated with narrower aneurysm necks (median 7 mm [IQR: 6.0–10.0] vs. 12.6 mm [IQR: 11.0–15.1], p = 0.023). Initial total aneurysm volume was lower in occluded aneurysms (2.6 cm^3^ [IQR: 1.9–4.9] vs. 10.6 cm^3^ [IQR: 5.5–13.9], p = 0.04). Neither time to occlusion nor rate of complete occlusion showed a statistically significant difference among saccular, dissecting, and fusiform aneurysms. Other analyzed variables did not show any statistically significant association with the assessed outcome variables (Supplementary Table 4).

Among the 12 aneurysms with pretreatment T1-Gd imaging, all showed wall enhancement and enhancement resolved after flow diversion in 4 (33%), all of which were completely occluded at last follow-up.

## 4 Discussion

This study examined the volumetric evolution and predictors of occlusion after flow diversion in a predominantly saccular anterior circulation cohort of large and giant aneurysms. We found that total aneurysm volume decreased by a median of 28% over a median follow-up of approximately 41 months, but this reduction was not statistically significant, reflecting both the modest sample and the heterogeneous volumetric response. The patent aneurysm volume, however, decreased to near elimination of the patent lumen. This is consistent with the 79% complete-occlusion rate (19 of 24 aneurysms), or 86% (19 of 22 aneurysms) when the two patients who died within weeks and never underwent follow-up angiography are excluded. However, volume evolution over time was heterogeneous, with some aneurysms growing and some shrinking, without identifiable predictive factors of this evolution. Narrower aneurysm necks and smaller initial total volumes were associated with complete occlusion, whereas age, sex, aneurysm type, and previous treatment were not.

Our findings align with prior reports documenting flow diversion-induced volume reduction, thrombus organization, and shrinkage of large and giant aneurysms in most cases (Sirakova et al., 2022). However, statistically significant reduction occurred only for patent (not total) volume, differing from Sirakova et al., who reported higher complete occlusion (our study 79% vs. 86%) and shrinkage rates (present study 75% vs. 89%) in exclusively giant (>25 mm) aneurysms (Sirakova et al., 2022). Wang et al. reported a similar proportion of aneurysms reducing in size (76%) in a cohort of unruptured large and giant aneurysms treated with flow diversion plus adjunctive coiling (Wang et al., 2019). Key differences from our study include their exclusion of asymptomatic, previously ruptured or treated, and fusiform/serpentine aneurysms, their analysis of total volume only, and greater use of multiple devices than in our cohort (present study 13% vs. 35%) (Wang et al., 2019). Yet, these results should be interpreted cautiously when compared with previous series. Published cohorts differ substantially in aneurysm size, symptoms, prior treatments, use of adjunctive coiling, imaging methodology, and follow-up duration (Wang et al., 2019; Sirakova et al., 2022; Carneiro et al., 2014). Such differences may account for variation in both reported occlusion rates and the degree of aneurysm shrinkage observed after treatment.

While volume reduction after treatment is a desirable process, it seems to be non-linear and poorly predictable, and is likely to be influenced by biological and hemodynamic factors intrinsic to the patient and aneurysm. Notably, Kuzmik et al. emphasized that similar aneurysms may have variable outcomes post-flow diversion, potentially due to differences in stent placement, device type, and patient-specific factors (Szikora et al., 2013; Kuzmik et al., 2013). Indeed, initial enlargement of aneurysm treated with FD is a well-recognized scenario (Suzuki et al., 2020; Carneiro et al., 2014). In our series some cases with complete occlusion showed larger final total volumes than initially, but all were smaller than interim volumes, indicating an initial enlargement phase due to clot formation preceding stable reduction (Sirakova et al., 2022). Volumes continued growing only in incompletely occluded cases, possible likely due to persistent endoleak (Carneiro et al., 2014) or the intrinsic biological changes of the aneurysm wall.

Relief of mass-effect symptoms after flow diversion has been linked to aneurysm shrinkage, and adjunctive coiling may promote organized thrombosis and higher occlusion rates than flow diversion alone (pooled OR 1.59, 95% CI 1.06–2.40) without excess complications (Wang et al., 2019; Kaiser et al., 2023; Abo Kasem et al., 2025). Our study was not designed to assess symptom relief, and symptom follow-up was incompletely recorded. Mechanistically, patent-volume reduction likely represents a key early step toward thrombus organization, occlusion, and eventual sac shrinkage. Antiplatelet therapy may also influence the timing of thrombosis and the pace of aneurysm remodeling. In contemporary flow-diversion practice, DAPT is commonly maintained for 3 to 6 months, after which treatment is often de-escalated to aspirin monotherapy if angiographic evolution is satisfactory (Flynn et al., 2026; Cagnazzo et al., 2019; Wang et al., 2025). However, these strategies remain heterogeneous across centers, and the present retrospective cohort was not designed to compare regimens (Ma et al., 2026).

Despite high efficacy of FD in the treatment of large and giant aneurysms, it remains intrinsically a high-risk procedure with reported major morbidity rates of 5–15% and mortality of 3–10% (Zhou et al., 2017; Kaiser et al., 2023). Adverse events such as post-FD inflammation, transient sac enlargement, distal ischemia, and hemorrhagic complications (including delayed rupture at 1.7–3%) are well-described, often unpredictable, and with poorly understood mechanisms (Kulcsar et al., 2011; Zhou et al., 2017; Brinjikji et al., 2015). These considerations, together with the need for prolonged DAPT support individualized follow-up strategies and cautious clinical decision-making. In our cohort, mortality was higher than previously reported: four of 24 patients (17%) died, and three of these died of post-treatment rupture of the treated, initially unruptured aneurysm. This likely reflects the predominance of large and giant lesions, the inclusion of previously treated aneurysms, and the small sample, but it underscores that flow diversion of these aneurysms carries a substantial and sometimes fatal early rupture risk that warrants explicit counseling and close early surveillance.

Despite notable progress in understanding aneurysm remodeling after FD, prediction of radiological or clinical evolution in large and giant aneurysms remains elusive (Wang et al., 2019; Sirakova et al., 2022; Carneiro et al., 2014; Meyer et al., 2024). Our follow-up data further suggest that aneurysm remodeling after flow diversion may extend well beyond the first post-treatment angiographic control. Although most occlusions were documented at 6 to 9 months, a minority of aneurysms required 24 to 45 months to be completely occluded. Clinical decisions therefore may rely on serial imaging and individualized risk assessment, with particular attention to neck size as a potential predictor of occlusion. Our findings highlight the value of regular volumetric monitoring by DSA or MRI to guide therapeutic decisions.

## 5 Limitations

This study must be interpreted in the context of its limitations. It is a retrospective, single center analysis with a modest sample size. Data regarding initial symptom evolution were suboptimally recorded and could not be extracted for this analysis. The risk of selection bias derived from excluding patients with no follow-up image is attenuated by the inclusion of consecutive cases with relatively broad eligibility criteria. Nonetheless, missing data and heterogeneity in imaging protocols and follow-up intervals may affect the generalizability of our findings. In addition, TOF sequence was not available to calculate aneurysm volume in four patients (cases 9,12,13 and 14) and T2 void was used instead. A sensitivity analysis excluding these cases showed no relevant changes in the overall findings. Finally, we included previously treated aneurysms which may increase the unpredictability of aneurysm occlusion while increasing the heterogeneity of the included cohort. These factors limit causal inference but reflect real-world nature of the analyzed cohort. Despite the aforementioned limitations, our dataset provides valuable insights into the complex volumetric response of large and giant aneurysms to flow diversion and identifies potential key morphological predictors of volume evolution.

## 6 Conclusion

Our findings suggest that flow diversion results in substantial patent volume reduction and high rates of complete occlusion for intracranial aneurysms 15 mm or larger, particularly those with narrow necks. Total volume reduction and aneurysm sac shrinkage might be expected in most cases, but the speed and pattern of this response seems to be unpredictable. Complications and outcome variability argue for individualized risk assessment and vigilant monitoring after treatment. Larger studies are warranted to refine volumetric predictors and optimize post-treatment management.

## Funding and disclosures

Independent funding support has been received from Helsinki University Hospital (State funding, Finland); Finska Läkaresällskapet; Medicinska Understödsföreningen Liv &amp; Hälsa; Svenska Kulturfonden, Maire Taponen Foundation and Waldemar von Frenckell’s Foundation. The funders had no role in study design, data collection, data analysis, data interpretation, or writing of the manuscript. The first and last authors had full access to all the data in the study and had final responsibility for the decision to submit for publication.

## Ethical approval and informed consent statements

The study was approved by the institutional review board (HUS/313/2022). In accordance with national regulations, informed consent was not required due to the retrospective, observational nature of the research. Patient confidentiality has been maintained throughout.

## Data availability

All data is anonymously presented in the manuscript. Further information can be provided upon request.

## Declaration of competing interest

The authors declare that they have no known competing financial interests or personal relationships that could have appeared to influence the work reported in this paper.
