# Supplementary material for: Longitudinal volume evolution of large and giant intracranial aneurysms following endovascular flow diversion
Source: Brain Spine. 2026 Jul 23;6:106172. doi: 10.1016/j.bas.2026.106172 (PMC13453463; doi:10.1016/j.bas.2026.106172)
Supplement: Supplementary file 1 [file mmc1.docx]

Supplementary Image 1. Bland-Altman plots illustrating interrater agreement for every analyzed volume at each time point. The mean interobserver difference was -0.015 for initial total volume, -0.005 for initial patent volume, 0.047 for intermediate total volume, 0.028 for intermediate patent volume, 0.126 for final total volume, and 0.018 for final patent volume.
