# Supplementary material for: Longitudinal volume evolution of large and giant intracranial aneurysms following endovascular flow diversion
Source: Brain Spine. 2026 Jul 23;6:106172. doi: 10.1016/j.bas.2026.106172 (PMC13453463; doi:10.1016/j.bas.2026.106172)
Supplement: Supplementary file 2 [file mmc2.docx]

**Supplementary Table 1**. Patient baseline characteristics.

| **Case** | **Age (y)** | **Sex** | **Symptoms** | **Hypertension** | **Smoker** | **Ruptured** | **mRS intial** |
| --- | --- | --- | --- | --- | --- | --- | --- |
| **1** | 66 | F | Diplopia(CN6) | N | N | N | 1 |
| **2** | 69 | F | Flicker | Y | N | N | 1 |
| **3** | 57 | M | Hypopituitarism | N | N | N | 2 |
| **4** | 63 | M | Hydrocephalus | N | N | N | 2 |
| **5** | 36 | M | Incidental | N | N | N | 0 |
| **6** | 66 | M | Incidental | N | N | N | 0 |
| **7** | 61 | F | Incidental | Y | N | N | 0 |
| **8** | 61 | F | Unrelated SAH | Y | Y (Previous) | N | 0 |
| **9** | 42 | M | Unrelated SAH | N | Y (Previous | N | 0 |
| **10** | 52 | M | Related SAH | N | Y (Previous) | Y | 5 |
| **11** | 73 | F | Visual Loss | N | N | N | 2 |
| **12** | 56 | F | Incidental | N | N | N | 0 |
| **13** | 68 | F | Diplopia (CN6) | Y | Y | N | 1 |
| **14** | 69 | M | Incidental | N | N | N | 1 |
| **15** | 74 | F | Incidental | N | N | N | 0 |
| **16** | 75 | F | Diplopia (CN3) | Y | N | N | 1 |
| **17** | 65 | M | Incidental | Y | Y | N | 1 |
| **18** | 47 | M | Incidental | N | N | N | 0 |
| **19** | 56 | F | Incidental | Y | Y | N | 1 |
| **20** | 59 | F | Diplopia (CN6) | Y | N | N | 1 |
| **21** | 72 | F | Incidental | N | N | N | 0 |
| **22** | 71 | M | Incidental | N | N | N | 1 |
| **23** | 63 | M | Stroke/TIA | N | Y | N | 1 |
| **24** | 33 | F | Incidental | N | N | N | 0 |

*Abbreviations*: CN: Cranial Nerve, F: Female, M: Male, mRS initial: Modified Rankin Scale at the moment of treatment, N: No, y: years, Y: Yes.
