# Supplementary material for: Longitudinal volume evolution of large and giant intracranial aneurysms following endovascular flow diversion
Source: Brain Spine. 2026 Jul 23;6:106172. doi: 10.1016/j.bas.2026.106172 (PMC13453463; doi:10.1016/j.bas.2026.106172)
Supplement: Supplementary file 3 [file mmc3.docx]

**Supplementary Table 2**. Sensitivity analysis comparing the different assessed volumes considering two cohorts, one including all patients and another one excluding the four patients in which TOF was not available.

| **Volume** | **Median** | | **Change** | **Mean** | | **Change** |
| --- | --- | --- | --- | --- | --- | --- |
|  | All | Reduced |  | All | Reduced |  |
| Total Initial | 3.50 | 4.50 | 1.00 | 5.72 | 6.40 | 0.68 |
| Total Interim | 2.80 | 8.35 | 5.55 | 9.23 | 11.80 | 2.57 |
| Total Final | 2.30 | 2.60 | 0.30 | 6.60 | 7.61 | 1.01 |
| Patent Initial | 2.00 | 2.40 | 0.40 | 2.50 | 2.81 | 0.31 |
| Patent Interim | 0.10 | 0.30 | 0.20 | 1.13 | 1.38 | 0.25 |
| Patent Final | 0.00 | 0.000 | 0.00 | 0.59 | 0.70 | 0.11 |
