# Supplementary material for: Longitudinal volume evolution of large and giant intracranial aneurysms following endovascular flow diversion
Source: Brain Spine. 2026 Jul 23;6:106172. doi: 10.1016/j.bas.2026.106172 (PMC13453463; doi:10.1016/j.bas.2026.106172)
Supplement: Supplementary file 4 [file mmc4.docx]

**Supplementary Table 3**. Characteristics of the treated aneurysms and outcomes.

| **Case** | **Type** | **Location** | **Rec** | **Morphometric (mm)** | | | **OKM filling scale** | | | **Time to Occlusion (m)** | **Last mRS** | **mRS change** | **Dead** |
| --- | --- | --- | --- | --- | --- | --- | --- | --- | --- | --- | --- | --- | --- |
|  |  |  |  | **Neck** | **Dome** | **Height** | **Initial** | **Interim** | **Final** |  |  |  |  |
| **1** | S | Cv | N | 12.0 | 13.0 | 17.0 | C |  | D | 7 | 1 | 0 | N |
| **2** | S | POph | N | 11.0 | 19.0 | 19.0 | B |  |  |  | 6 | +5 | Y |
| **3** | S | Cv | Y | 12.6 | 7.2 | 7.7 | B |  | B | 100 | 2 | 0 | N |
| **4** | S | Cv | N | 10.0 | 14.0 | 19.6 | B | D | D | 25 | 1 | -1 | N |
| **5** | S | Cv | N | 6.3 | 8.0 | 16.7 | B |  | D | 8 | 0 | 0 | N |
| **6** | Fs | Basilar | N | 6.0 | 12.0 | 16.0 | C | D | D | 7 | 0 | 0 | N |
| **7** | S | Cv | N | 8.9 | 23.3 | 22.8 | D |  | D | 6 | 0 | 0 | N |
| **8** | S | Term | Y | 5.4 | 15.5 | 15.1 | C | C | D | 9 | 0 | 0 | N |
| **9** | S | Term | Y | 7.2 | 15.1 | 12.7 | C | C | D | 6 | 0 | 0 | N |
| **10** | Fs | Basilar | N | 15.0 | 25.0 | 15.0 | C | C | D | 6 | 6 | +1 | Y |
| **11** | S | Cv | N | 11.0 | 26.0 | 21.0 | B | B | B | 100 | 2 | 0 | N |
| **12** | S | Poph | N | 6.7 | 12.0 | 17.0 | B |  | D | 9 | 0 | 0 | N |
| **13** | D | Cv | N | 4.0 | 10.0 | 18.0 | B | D | D | 6 | 0 | -1 | N |
| **14** | S | Term-A1 | Y | 7.0 | 14.0 | 19.0 | C | C | D | 45 | 1 | 0 | N |
| **15** | S | Cv | N | 19.0 | 30.0 | 28.0 | C | C | C | 45 | 1 | 1 | N |
| **16** | D | Cv | N | 15.0 | 33.0 | 23.7 | B |  | D | 7 | 1 | 1 | N |
| **17** | D | Term | Y | 5.6 | 16.0 | 10.0 | B | C | D | 6 | 0 | -1 | N |
| **18** | S | POph | N | 5.6 | 15.0 | 13.6 | B |  | D | 24 | 0 | 0 | N |
| **19** | D | Basilar | N | 9.0 | 17.0 | 21.0 | B |  |  |  | 6 | +5 | Y |
| **20** | S | Cv | N | 10.5 | 29.4 | 21.0 | C | D | D | 7 | 0 | -1 | N |
| **21** | S | Term | N | 8.5 | 19.0 | 11.5 | C |  | D | 24 | 0 | 0 | N |
| **22** | S | Term | Y | 5.7 | 12.4 | 15.1 | B | D | D | 6 | 6 | +5 | Y |
| **23** | S | Term-A1 | N | 3.4 | 17.0 | 42.0 | D |  | D | 6 | 1 | 0 | N |
| **24** | S | Cv | N | 7.3 | 16.0 | 11.1 | B |  | D | 7 | 0 | 0 | N |

Cv: Cavernous segment Internal Carotid Artery, D: Dissecting, Fs: Fusiform, m: months, mRS last: Modified Rankin Scale at last follow-up, N: No, OKM: O’Kelly-Marotta Scale, POph: Paraopthtalmic segment Internal Carotid Artery, R: Recurrent, S: Sacular, , Term: Terminal or Communicating segment Internal Carotid artery, Uk: Unknown, Y: Yes.
