# Supplementary material for: Longitudinal volume evolution of large and giant intracranial aneurysms following endovascular flow diversion
Source: Brain Spine. 2026 Jul 23;6:106172. doi: 10.1016/j.bas.2026.106172 (PMC13453463; doi:10.1016/j.bas.2026.106172)
Supplement: Supplementary file 5 [file mmc5.docx]

**Supplementary Table 4:** Studied variables, outcomes and results of their statistical comparison.

| **Variable** | **Outcome** | **Test** | **N** | **Statistic** | **P-value** |
| --- | --- | --- | --- | --- | --- |
| AP regime | Time to Occlusion | *Spearman correlation* | 24 | 0.201 | 0.369 |
| AP regime | Last OKM (ordinal) | *Spearman correlation* | 24 | -0.144 | 0.522 |
| AP regime | Last OKM (A-B vs C-D) | *Mann-Whitney U* | 24 | 21.0 | 0.948 |
| AP regime | Complete Occlusion | *Mann-Whitney U* | 24 | 41.0 | 0.650 |
| Age | Time to Occlusion | *Spearman correlation* | 24 | 0.163 | 0.469 |
| Age | Last OKM (ordinal) | *Spearman correlation* | 24 | -0.267 | 0.230 |
| Age | Last OKM (A-B vs C-D) | *Mann-Whitney U* | 24 | 24.0 | 0.701 |
| Age | Complete Occlusion | *Mann-Whitney U* | 24 | 45.0 | 0.484 |
| An. Dome | Time to Occlusion | *Spearman correlation* | 24 | -0.061 | 0.786 |
| An. Dome | Last OKM (ordinal) | *Spearman correlation* | 24 | -0.115 | 0.611 |
| An. Dome | Last OKM (A-B vs C-D) | *Mann-Whitney U* | 24 | 17.0 | 0.775 |
| An. Dome | Complete Occlusion | *Mann-Whitney U* | 24 | 45.5 | 0.443 |
| An. Height | Time to Occlusion | *Spearman correlation* | 24 | 0.036 | 0.875 |
| An. Height | Last OKM (ordinal) | *Spearman correlation* | 24 | -0.082 | 0.717 |
| An. Height | Last OKM (A-B vs C-D) | *Mann-Whitney U* | 24 | 15.5 | 0.648 |
| An. Height | Complete Occlusion | *Mann-Whitney U* | 24 | 46.0 | 0.418 |
| An. Location | Time to Occlusion | *Insufficient data* | 24 | - | - |
| An. Location | Last OKM (ordinal) | *Insufficient data* | 24 | - | - |
| An. Location | Last OKM (A-B vs C-D) | *Insufficient data* | 24 | - | - |
| An. Location | Complete Occlusion | *Insufficient data* | 24 | - | - |
| An. Max Size | Time to Occlusion | *Spearman correlation* | 24 | 0.014 | 0.949 |
| An. Max Size | Last OKM (ordinal) | *Spearman correlation* | 24 | -0.224 | 0.317 |
| An. Max Size | Last OKM (A-B vs C-D) | *Mann-Whitney U* | 24 | 22.0 | 0.864 |
| An. Max Size | Complete Occlusion | *Mann-Whitney U* | 24 | 51.0 | 0.216 |
| An. Neck | Time to Occlusion | *Spearman correlation* | 24 | 0.343 | 0.119 |
| An. Neck | Last OKM (ordinal) | *Spearman correlation* | 24 | -0.482 | **0.023** |
| An. Neck | Last OKM (A-B vs C-D) | *Mann-Whitney U* | 24 | 33.0 | 0.153 |
| An. Neck | Complete Occlusion | *Mann-Whitney U* | 24 | 62.0 | **0.030** |
| Edema | Time to Occlusion | *Mann-Whitney U* | 18 | 44.5 | 0.442 |
| Edema | Last OKM (ordinal) | *Mann-Whitney U* | 18 | 27.0 | 0.220 |
| Edema | Last OKM (A-B vs C-D) | *Fisher exact* | 18 | inf | 0.529 |
| Edema | Complete Occlusion | *Fisher exact* | 18 | 1.67 | 1.000 |
| FD Diameter | Time to Occlusion | *Spearman correlation* | 24 | 0.041 | 0.856 |
| FD Diameter | Last OKM (ordinal) | *Spearman correlation* | 24 | -0.014 | 0.949 |
| FD Diameter | Last OKM (A-B vs C-D) | *Mann-Whitney U* | 24 | 17.5 | 0.782 |
| FD Diameter | Complete Occlusion | *Mann-Whitney U* | 24 | 40.5 | 0.680 |
| FD Length | Time to Occlusion | *Spearman correlation* | 24 | 0.074 | 0.744 |
| FD Length | Last OKM (ordinal) | *Spearman correlation* | 24 | -0.215 | 0.337 |
| FD Length | Last OKM (A-B vs C-D) | *Mann-Whitney U* | 24 | 23.0 | 0.767 |
| FD Length | Complete Occlusion | *Mann-Whitney U* | 24 | 43.0 | 0.564 |
| FD type | Time to Occlusion | *Insufficient data* | 24 | - | - |
| FD type | Last OKM (ordinal) | *Insufficient data* | 24 | - | - |
| FD type | Last OKM (A-B vs C-D) | *Insufficient data* | 24 | - | - |
| FD type | Complete Occlusion | *Insufficient data* | 24 | - | - |
| Immediate OKM | Time to Occlusion | *Spearman correlation* | 24 | -0.241 | 0.280 |
| Immediate OKM | Last OKM (ordinal) | *Spearman correlation* | 24 | 0.172 | 0.445 |
| Immediate OKM | Last OKM (A-B vs C-D) | *Mann-Whitney U* | 24 | 9.0 | 0.182 |
| Immediate OKM | Complete Occlusion | *Mann-Whitney U* | 24 | 26.0 | 0.363 |
| Initial GD enh | Time to Occlusion | *Insufficient data* | 10 | - | - |
| Initial GD enh | Last OKM (ordinal) | *Insufficient data* | 10 | - | - |
| Initial GD enh | Last OKM (A-B vs C-D) | *Insufficient data* | 10 | - | - |
| Initial GD enh | Complete Occlusion | *Insufficient data* | 10 | - | - |
| Initial Patent Volume | Time to Occlusion | *Spearman correlation* | 24 | 0.199 | 0.375 |
| Initial Patent Volume | Last OKM (ordinal) | *Spearman correlation* | 24 | -0.384 | 0.077 |
| Initial Patent Volume | Last OKM (A-B vs C-D) | *Mann-Whitney U* | 24 | 32.0 | 0.216 |
| Initial Patent Volume | Complete Occlusion | *Mann-Whitney U* | 24 | 54.0 | 0.141 |
| Initial TOF HI | Time to Occlusion | *Mann-Whitney U* | 12 | 9.5 | 1.000 |
| Initial TOF HI | Last OKM (ordinal) | *Mann-Whitney U* | 12 | 8.0 | 0.671 |
| Initial TOF HI | Last OKM (A-B vs C-D) | *Fisher exact* | 12 | 0.00 | 1.000 |
| Initial TOF HI | Complete Occlusion | *Fisher exact* | 12 | 2.67 | 1.000 |
| Initial Total Volume | Time to Occlusion | *Spearman correlation* | 24 | -0.094 | 0.676 |
| Initial Total Volume | Last OKM (ordinal) | *Spearman correlation* | 24 | -0.447 | **0.037** |
| Initial Total Volume | Last OKM (A-B vs C-D) | *Mann-Whitney U* | 24 | 25.0 | 0.606 |
| Initial Total Volume | Complete Occlusion | *Mann-Whitney U* | 24 | 50.0 | **0.040** |
| Recurrent An | Time to Occlusion | *Mann-Whitney U* | 24 | 49.5 | 0.940 |
| Recurrent An | Last OKM (ordinal) | *Mann-Whitney U* | 24 | 50.5 | 0.805 |
| Recurrent An | Last OKM (A-B vs C-D) | *Fisher exact* | 24 | 0.33 | 0.481 |
| Recurrent An | Complete Occlusion | *Fisher exact* | 24 | 0.86 | 1.000 |
| Sex | Time to Occlusion | *Mann-Whitney U* | 24 | 68.5 | 0.614 |
| Sex | Last OKM (ordinal) | *Mann-Whitney U* | 24 | 55.5 | 0.620 |
| Sex | Last OKM (A-B vs C-D) | *Fisher exact* | 24 | 1.00 | 1.000 |
| Sex | Complete Occlusion | *Fisher exact* | 24 | 3.00 | 0.594 |

An: Aneurysm, AP: Antiplatelet, Enh: Enhancement, FD: Flow Diverter, GD: Gadolinium, HI: Hyperintensity, OKM: O´Kelly Marotta scale.

**Note:** P-values <0.05 shown in bold. Tests selected based on variable type: Spearman correlation (continuous/ordinal predictors vs ordinal outcomes), Mann-Whitney U (binary predictors vs ordinal outcomes, or continuous outcomes vs binary predictors), Fisher exact test (binary predictors vs binary outcomes). "Insufficient data" indicates N<10, no variability, or categorical variables with multiple levels.
